# Chemical structures, biological activities, and medicinal potentials of amine compounds detected from *Aloe* species

**DOI:** 10.3389/fchem.2024.1363066

**Published:** 2024-03-01

**Authors:** Adamu Tizazu Yadeta

**Affiliations:** Department of Chemistry, College of Natural and Computational Sciences, Mekdela Amba University, Tulu Awuliya, Ethiopia

**Keywords:** *Aloe* species, nitrogen, amines, biological activities, medicinal potentials

## Abstract

Unrestricted interest in *Aloe* species has grown rapidly, and a lot of research is currently being done to learn more about the properties of the various *Aloe* constituents. Organic compounds containing amine as functional group are present in a vivid variety of compounds, namely, amino acids, hormones, neurotransmitters, DNA, alkaloids, dyes, etc. These compounds have amine functional groups that have various biological activities, which make them responsible for medicinal potential in the form of pharmaceutical, nutraceutical, and cosmeceutical applications. Consequently, the present review work provides an indication of the amines investigated in *Aloe* species and their therapeutic uses. Various amine compounds of the *Aloe* species have effective biological properties to treat diseases. Generally, the genus *Aloe* has various active amine-containing compounds to combat diseases when humans use them in various forms.

## 1 Introduction

Unrestricted interest in *Aloe* species has grown rapidly and a lot of research is currently being done to learn more about the properties of the various *Aloe* constituents (Singab et al., 2015). *Aloe* plants are a unique source of phytochemicals because they can tolerate hot, dry weather. As a result, they store water and vital chemical components in their swollen, succulent leaves (Wójcik et al., 2021). Numerous studies conducted both *in vitro* and *in vivo* have confirmed the biological properties of *Aloe* species, including wound healing, anti-tumoral, anti-inflammatory, antimicrobial, antimalarial, anticancer, etc. properties. Mostly, these characteristics could not be ascribed to a single class of compounds, but rather to a variety of compounds found in the phytochemical profile of *Aloe* extracts (Hamman, 2008; Sharrif Moghaddasi and Res, 2011; Andrea et al., 2020). Alkaloids, amino acids, vitamins, hormones, proteins, polyphenols, saccharides, organic acids, and other naturally occurring phytochemicals are abundant in *Aloe species* (Zayed et al., 2012; Ufnal et al., 2015; Sánchez et al., 2020). Most of these phytochemicals have the functional group amine. Amines are present in large amounts in all living things (Kaur et al., 2018).

**FIGURE 1:**
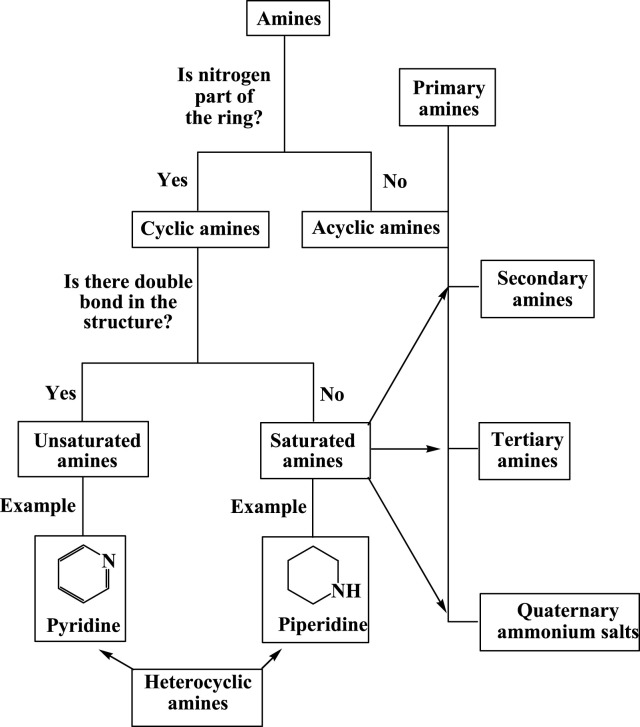
Classification of amines.

Amines are nitrogen-containing functional groups of organic chemistry that arise from the substitution of an alkyl or aryl group for one or more hydrogen atoms in ammonia (NH_3_) (Lawrence, 2004). Amines are categorized as primary (RNH_2_), secondary (R_2_NH), or tertiary (R_3_N) depending on whether one, two, or three of the hydrogen atoms in ammonia have been substituted out for organic groups (Graton et al., 2005). Quaternary ammonium compounds fall into a fourth category. They are created by substituting all four hydrogen atoms in the ammonium ion, NH_4_^+^; an anion (R_4_N^+^X^−^) is required in this case (Grossman and Grossman, 2003). When nitrogen is part of the ring, the compound is known as heterocyclic amine; each heterocyclic ring system has a unique parent name. Always, the nitrogen atom is assigned to position 1 in heterocyclic amines (Jacobi, 2018). Compounds such as pyridine, pyrrole, quinoline, imidazole, indole, pyrimidine, pyrrolidine, and piperidine are examples of commonly known heterocyclic amines. These amines are used as parent names for their substituents (Li, 2013). Generally, amines have two broad categories: cyclic amines and acyclic amines. Cyclic amines are heterocyclic amines that have one or more nitrogen as heteroatoms. Heterocyclic amines have no primary amines due to their rings. Cyclic amines are further classified into saturated and unsaturated amines. Unsaturated amines which have C^_^N double bonds cannot be primary, secondary, etc. In another way, acyclic amines have nitrogen out of the ring. Moreover, any amine falls into the categories of heterocyclic amine, primary amine, secondary amine, tertiary amine, or quaternary ammonium salt (Figure 1).

Organic compounds containing amine as functional group are present in a vivid variety of compounds, namely, amino acids, hormones, neurotransmitters, DNA, alkaloids, dyes, etc. Drugs such as morphine, nicotine, codeine, and heroin, etc., that have physiological properties in humans also contain amino groups in one form or another. Amines are basic because of the presence of a lone pair of electrons on nitrogen (El-Sakka, 2010; Otimenyin, 2022). Amine-containing compounds have unique properties that make them biologically active molecules. A lone pair of electrons on nitrogen dominates the chemistry of amines, making them both bases and nucleophiles (Morgenthaler et al., 2007; Vedejs and Denmark, 2016). Due to these properties, the compounds that have amine functional groups have various biological activities that are responsible for pharmaceutical, nutraceutical, and cosmeceutical applications. Using amines for a variety of applications needs attention currently. However, to the best of my knowledge, no comprehensive work was done regarding the amine-containing compounds detected in the *Aloe* species for their medicinal potential. Consequently, the present work summarizes amine-containing compounds investigated from *Aloe* species and their therapeutic uses. More generally, the core point of the current review is to describe the chemical structure of the detected amine compounds from *Aloe* species and relate them to the studied medicinal activities of the plants.

## 2 The review methodology

The relevant sources for this study were retrieved using search engines including Google Scholar, PubMed, and Science Direct. For the purpose of finding relevant sources, several combinations of terms and phrases such as amines, alkaloids, amino acids, vitamins, hormones, *Aloe* species, *Aloe* species phytochemicals, *Aloe* species applications, and medicinal activities of *Aloe* extracts and compounds were utilized. This review covered studies on chemical structures of amines analyzed from *Aloe* species, the medicinal potential of *Aloe* extracts, and amine compounds. Reports on such activities based on other than amine compounds were excluded. Studies that were published in languages other than English were not at all taken into account. Following the collection of all sources, a rapid study of the sources’ titles, abstracts, and conclusions was done to determine which ones met the qualifying requirements. The chosen sources were then carefully examined in order to prepare this review paper. The chemical structures of the compounds were depicted using ChemDraw Ultra 8.0 software, while citations and checking references were provided using Mendeley Desktop software.

## 3 Structures of amines detected from *Aloe* species

### 3.1 Alkaloids

The name alkaloid (“alkali-like”) was originally applied to the substances because, like the inorganic alkalis, they react with acids to form salts (Alamgir, 2018). Alkaloids are small organic molecules that contain nitrogen, usually in a ring. Therefore, alkaloids are one of the heterocyclic amine compounds having nitrogen as a heteroatom (Bhardwaj et al., 2021). Alkaloids are an important group of biologically active amines that are mostly synthesized by plants to protect them from being eaten by insects and other animals. Alkaloids in nature are vast, so we need to classify them into specific groups. Compounds such as nucleosides, vitamins, hormones, some amino acids, etc. are alkaloids. For instance, pyridoxine is a vitamin as well as an alkaloid; proline is α-amino acid as well as an alkaloid. However, such compounds are together called amines (Wade, 2013; Mcmurry, 2016).

**FIGURE 2:**
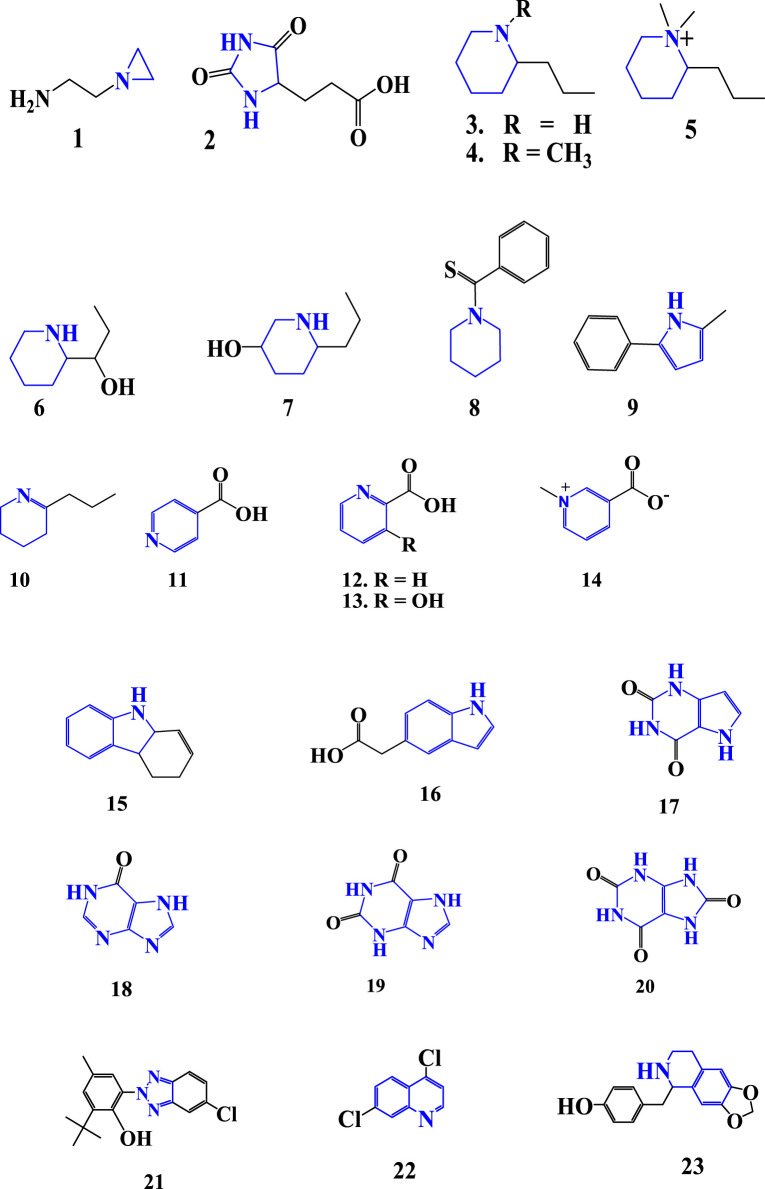
Structure of alkaloids detected from *Aloe* species.

Alkaloids have been analyzed from *Aloe* species quantitatively and qualitatively (Sonam and Archana, 2016; Usman et al., 2020). Phytochemical screening showed the presence of alkaloids in various *Aloe* species such as *A. adigratana*, *A. barbadensis*, *A. calidophila*, *A. ferox*, *A. vera*, *A. turkanensis*, and *A. Gilbertii* from the leaves of plants such as gel, latex, skin, whole leaf (Salehi et al., 2018), roots (Jemal et al., 2018), and flowers (Martínez-Sánchez et al., 2020). Also, a number of alkaloid compounds have been isolated from *Aloe* species (Salehi et al., 2018). The detected alkaloids from the *Aloe* species are heterocyclic amines, and they are derivatives of the base heterocyclic amines (Table 1). The nomenclatures of some alkaloids were not copied directly from the names reported in the literature. Because some names of alkaloids are complex to write. For instance, the compound 23, which was obtained from GC-MS analysis of ethanolic extract *A. vera* was written in the literature as phenol, 4-[(5,6,7,8-tetrahydro-1,3-dioxolo [4,5-g]isoquinolin-5yl)methyl]. This name is too long; instead, ‘norcinnamolaurine’, the other name of the compound, was used in the current work (Table 1; Figure 2).

**TABLE 1:** Alkaloids of *Aloe* species.

No	Name	Detection method used	Molecular formula	Molecular weight (g/mol)	*Aloe* species	References
1	(2-aziridinylethyl) amine	GC-MS analysis	C_4_H_10_N_2_	86.14	*A.vera*	Alghamdi et al. (2023)
2	Hydantoinpropionic acid	GC-MS analysis	C_6_H_8_N_2_O_4_	172.14	*A. greatheadii*	Botes et al. (2008)
3	Coniine	Phytochemical investigations and GC-MS analysis	C_8_H_17_N	127.23	*A. sabaea, A. globuligemma, and A. viguieri*	Blitzke et al., 2000; Hotti et al., 2017
4	*N*-methylconiine	GC-MS analysis	C_9_H_19_N	141.25	*A. globuligemma* and *A. viguieri*	Hotti et al. (2017)
5	N,N-dimethylconiine	Phytochemical investigations	C_10_H_22_N	156.24	*A. sabaea*	Blitzke et al. (2000)
6	conhydrine	GC-MS analysis	C_8_H_15_NO	141.21	*A. ballyii*	Hotti et al. (2017)
7	Pseudoconhydrine	GC-MS analysis	C_8_H_17_NO	143.23	*A. deltoideodonta*	Hotti et al. (2017)
8	1-(phenylthioxomethyl) piperidine	GC-MS analysis	C_12_H_15_NS	205.32	*A. vera*	Bawankar et al. (2013)
9	2-methyl-5-phenyl-pyrrole	GC-MS analysis	C_11_H_11_N	157.21	*A.vera*	Alghamdi et al. (2023)
10	γ-coniceine	Phytochemical investigations and GC-MS analysis	C_8_H_15_N	125.21	*A. sabaea, A. globuligemma, and A. viguieri*	Blitzke et al., 2000; Hotti et al., 2017
11	Isonicotinic acid	GC-MS analysis	C_6_H_5_NO_2_	123.11	*A. greatheadii*	Botes et al. (2008)
12	Picolinic acid	GC-MS analysis	C_6_H_5_NO_2_	123.11	*A. vera*	Nejatzadeh-Barandozi (2013)
13	3-hydroxypicolinic acid	GC-MS analysis	C_6_H_5_NO_3_	139.12	*A. greatheadii*	Botes et al. (2008)
14	Trigonelline	^1^H NMR	C_7_H_7_NO_2_	137.14	*A. vera*	Martínez-Sánchez, et al. (2020)
15	Hexahydrobenzoindole	GC-MS analysis	C_12_H_13_N	171.24	*A. greatheadii*	Botes et al. (2008)
16	Indole-5-acetic acid	GC-MS analysis	C_10_H_9_NO_2_	180.21	*A. greatheadii and A ferox*	Botes et al., 2008; Loots et al., 2007
17	Pyrrolo [3,2-d]pyrimidin-2,4 (1H,3H)-dione	GC-MS analysis	C_6_H_5_N_3_O_2_	151.12	*A. vera*	Alghamdi et al. (2023)
18	Hypoxanthine	GC-MS analysis and HPLC	C_5_H_4_N_4_O	136.11	*A. greatheadii*	Botes et al., 2008; Salehi et al., 2018
19	Xanthine	HPLC	C_5_H_4_N_4_O_2_	152.11	*A. ferox*	Salehi et al. (2018)
20	Uric acid	-	C_5_H_4_N_4_O_3_	168.11	*A. vera*	Hamman (2008)
21	Bumetrizole	GC-MS analysis	C_17_H_18_ClN_3_O	315.80	*A. jucunda*	Dey et al. (2017)
22	4,7-dichloroquinoline	UV, NMR, and MS analyses	C_9_H_5_Cl_2_N	198.05	*A. hijazensis*	Abd-Alla et al. (2009)
23	Norcinnamolaurine	GC-MS analysis	C_17_H_17_NO_3_	283.32	*A. vera*	Alghamdi et al. (2023)

“ -” Unspecified.

### 3.2 Vitamins

Vitamins are organic compounds required by humans as nutrients in small amounts known as micronutrients. They are very important compounds for the activities of enzyme cofactors and coenzymes. The term vitamin is derived from the Latin words ‘vital’ and ‘amine’, combined as “vital amines” or “vitamines”. The “e" at the end of “vitamine” was later removed when it was realized that vitamins need not be nitrogen-containing amines. Therefore, not all vitamins are amines (Menon et al., 2008; Miodownik and Lerner, 2010; Godswill et al., 2020). Among the vitamins detected from *Aloe* species are choline, folic acid (B_9_), vitamin B_1_ (thiamine), niacin (nicotinic acid, also known as vitamin B_3_), vitamin B_2_ (riboflavin), vitamin B_6_ (pyridoxine), and vitamin B_12_ (cyanocobalamin). Although vitamin B_6_ is a collective term for pyridoxine, pyridoxal, and pyridoxamine, pyridoxine occurs predominantly in plants, whereas pyridoxal and pyridoxamine are found in foods obtained from animals (Hellmann and Mooney, 2010; Dewangan and Bhatia, 2023). Therefore, the B_6_ detected in *Aloe* plants is pyridoxine (**compound 28**). Choline is not strictly a vitamin but is an essential nutritional quaternary ammonium salt form of amine. However, it is known in the literature by means of vitamin B_4_ (Rakkar and Hillier, 2007). In *Aloe* species, it has been reported as a vitamin. Being mostly studied and known by various names, *A. vera* is the species for which these vitamins are reported (Akaberi et al., 2016; Mahor and Ali, 2016; Maan et al., 2018; Pegu and Sharma, 2019; Martínez-Sánchez et al., 2020; Adlakha et al., 2022).

**FIGURE 3:**
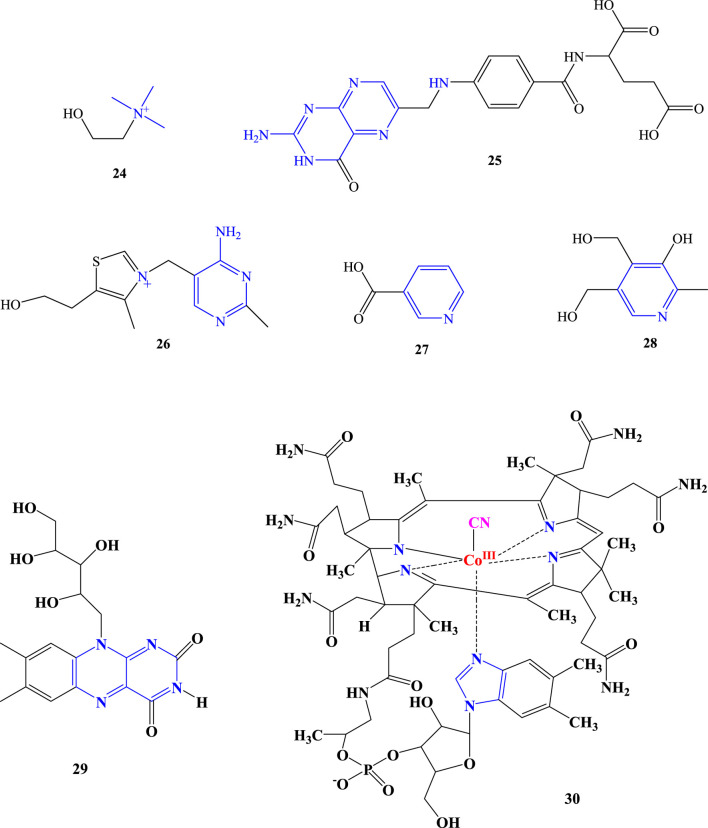
Amine containing vitamins detected from *Aloe* species.

The majority of vitamin studies omitted information about the analytical techniques used to detect them. Munoz et al. (2015) have reported that the Association of Official Analytical Chemists (AOAC) is utilized for the purpose of detecting vitamins in *A. vera*. Furthermore, choline from *A. vera* has been detected using ^1^H NMR (Martínez-Sánchez et al., 2020). Since nitrogen is a structural component of all these vitamins, they are all amine compounds (Figure 3). Because of this nitrogen, the vitamin’s amines can be classified as primary, secondary, tertiary, quaternary ammonium salts, and/or heterocyclic amines (Table 2).

**TABLE 2:** Vitamins detected in *Aloe* species.

No	Common names	Vitamin type	Amine type	Molecular formula	Molecular weight (g/mol)
24	Choline	Vitamin B_4_	Quaternary ammonium salts	C_5_H_14_NO^+^	104.17
25	Folic acid	Vitamin B_9_	1°, 2° and pteridine	C_19_H_19_N_7_O_6_	441.4
26	Thiamin	Vitamin B_1_	More than one type	C_12_H_17_N_4_OS^+^	265.36
27	Nicotinic acid	Vitamin B_3_	Pyridine	C_6_H_5_NO_2_	123.11
28	Pyridoxine	Vitamin B_6_	Pyridine	C_8_H_11_NO_3_	169.18
29	Riboflavin	Vitamin B_2_	Pteridine	C_17_H_20_N_4_O_6_	376.4
30	Cyanocobalamin	Vitamin B_12_	More than one type	C_63_H_88_CoN_14_O_14_P	1355.4

### 3.3 Hormones and neurotransmitters

**FIGURE 4:**
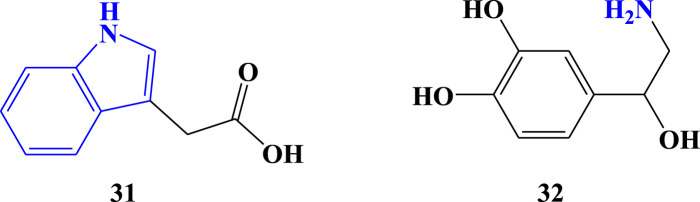
Amine hormones detected from *Aloe* species.

Auxins are a group of naturally occurring plant hormones (Agboola et al., 2014), and they are one of the two hormones repeatedly reported from *Aloe* species such as *A. vera* (Hamman, 2008). In other studies, indole-3-acetic acid (Figure 4; Compound 31) was detected in *A. vera (* Nejatzadeh-Barandozi, 2013) and *A ferox* (Loots et al., 2007). This indole-3-acetic acid is one of the auxin compounds (Santos et al., 2021). However, the works of literature that reported the presence of auxins from *Aloe* species have not mentioned indole-3-acetic acid presence as well, and the literature that reported indole-3-acetic acid has not explained it as one of auxin hormones. Therefore, the current work combines the literature to clarify the auxin hormones present in the *Aloe* species in the form of indole-3-acetic acid. The newly reported amine-containing hormone from *Aloe* species is noradrenaline (32, Figure 4). It was analyzed from *A. barbadensis* Mill. (Ahluwalia et al., 2022). Noradrenaline, also called norepinephrine, is an organic chemical in the catecholamine family that functions in the brain and body as both a hormone and neurotransmitter (Savaliya and Georrge, 2020). Hence, it is important to understand the two amine hormones of *Aloe* species, both, namely, and structurally.

### 3.4 Nucleobases, nucleosides, and nucleotides

**FIGURE 5:**
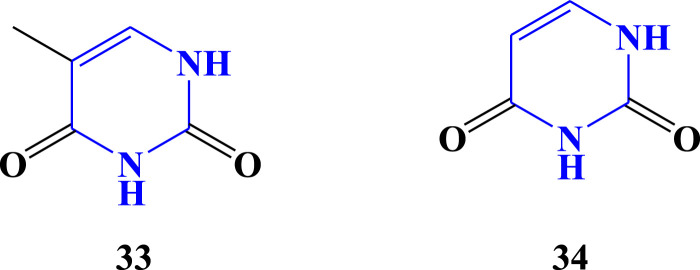
Nucleobases detected from *Aloe* species.

Nucleobases (nitrogenous bases or simply bases) are nitrogen-containing biological compounds that form nucleosides, which, in turn, are components of nucleotides, with all of these monomers constituting the basic building blocks of nucleic acids (Al-hayali, 2022). Among the five nucleobases, thymine and uracil (Figure 5) were detected in *A. vera* (Nejatzadeh-Barandozi, 2013), *A. ferox* (Loots et al., 2007), and *A. greatheadii* (Botes et al., 2008). These bases are pyrimidine bases because they are derived from pyrimidine. The main distinction between thymine (T) and uracil (U) lies in their chemical structure. Thymine **(33)** has a methyl group (CH_3_) attached to its ring structure, whereas uracil (**34**) does not have this methyl group. This structural difference is responsible for the various roles of thymine in DNA and uracil in RNA (Ono et al., 2011; Lippert and Sanz Miguel, 2016). In addition to nucleobases, nucleoside and nucleotide have been detected in *Aloe* species. Adenosine (nucleoside, **36**) and adenosine monophosphate (nucleotide, **37**) have been detected quantitatively from the flower of *A. vera* (Martínez-Sánchez et al., 2020).

### 3.5 Amino sugars and related amines

**FIGURE 6:**
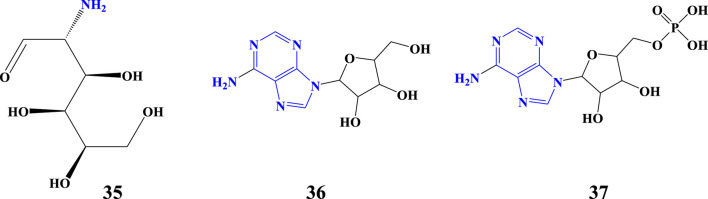
Glucosamine, adenosine, and AMP detected from *Aloe* species.

Amino sugars are chemical compounds with a sugar backbone where an amine group has taken the place of one of the hydroxyl groups (Parsons, 2021). Glucosamine (**35**) is an amino sugar and a prominent precursor in the biochemical synthesis of glycosylated proteins and lipids (Dalirfardouei et al., 2016). A sugar in which an amino group replaces the anomeric OH is called a glycosylamine. Adenosine (**36**) is glycosylamine, in which the amino component is a purine. The molecule consists of an adenine attached to a ribose via a β-N_9_-glycosidic bond. Adenosine is one of the four nucleoside building blocks of RNA (and its derivative, deoxyadenosine, is a building block of DNA), which are essential for all life on earth (Vázquez-Salazar et al., 2018; Rodrigues, 2021). A nucleotide consists of a sugar molecule (either ribose in RNA or deoxyribose in DNA) attached to a phosphate group and a nitrogen-containing base (Tripathi et al., 2023). Adenosine monophosphate (AMP), also known as 5′-adenylic acid, is a nucleotide. AMP (**37**) consists of a phosphate group, the sugar ribose, and the nucleobase adenine (Najeeb et al., 2023). Glucosamine, adenosine, and AMP have been detected in *Aloe* species (Martínez-Sánchez et al., 2020; Ahluwalia et al., 2022). These compounds have amine and sugar in their structure, which makes them similar to one another (Figure 6).

### 3.6 Amino acids

**FIGURE 7:**
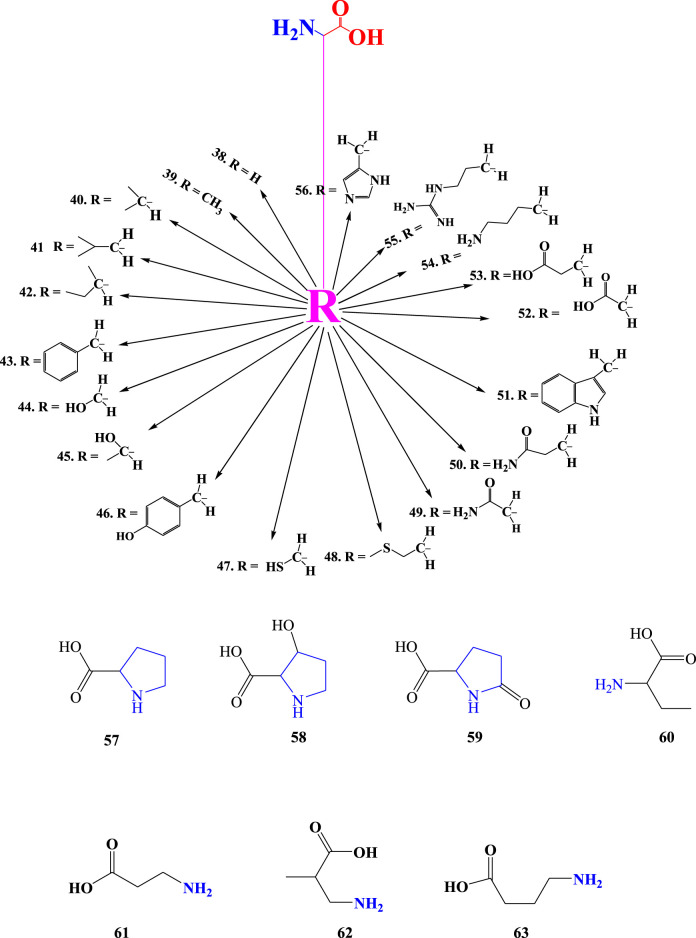
Amino acids detected from *Aloe* species.

Amino acid is any of a group of organic molecules that consist of a basic amino group (NH_2_), an acidic carboxyl group (COOH), and an organic side chain *(R* group, unique to each amino acid) (Taniguchi, 2010). Amino acids are primary amines (Mcmurry, 2016)**.** Amino acids are essential plant compounds serving as the building blocks of proteins, the predominant forms of nitrogen (N) distribution, and signaling molecules (Moe, 2013). The amino acids of *Aloe* plants have been reported by many authors in both quantitative and qualitative analyses. Although there are a number of methods to detect amino acids, many of the amino acids (Table 3) detected in *Aloe* species were using an amino acid analyzer equipped with an HPLC and UV detector (Kim et al., 2013; Chandra Sekhar Singh et al., 2018). In addition to that, HPLC and ^1^H NMR were used for the detection of amino acids in *Aloe* species (Hendrawati et al., 2019; Martínez-Sánchez et al., 2020). The analyzed amino acids from *Aloe* species are the twenty standard α-amino acids and their derivatives, which differ only in side chain (R) groups (Figure 7: structures 38–60). The noncyclic α-amino acids have an amino group in their C_2_, which ‘2-amino’ is common for all. In addition to α-amino acids, β-amino acids (**61** and **62**) and γ-amino acids (63) have been analyzed from *Aloe* species (Figure 7). Totally, the amino acids detected from *Aloe* species have been summarized in Table 3; Figure 7.

**TABLE 3:** Amino acids detected from *Aloe* species.

№	Original/source common name	IUPAC name	Molecular formula	Molecular weight (g/mol)	*Aloe* species	References
38	Glycine	2-aminoethanoic acid	C_2_H_5_NO_2_	75.07	*A. vera, A. saponaria, A. arborescens*	Kim et al. (2013); Mahor and Ali, (2016)
39	Alanine	2-aminopropanoic acid	C_3_H_7_NO_2_	89.09	*A. vera, A. saponaria,* *A. arborescens*	Kim et al. (2013); Martínez-Sánchez et al. (2020)
40	Valine	2-amino-3-methylbutanoic acids	C_5_H_11_NO_2_	117.15	*A. vera, A. saponaria,* and *A. arborescens*	Kim et al. (2013); Martínez-Sánchez et al. (2020)
41	Leucine	2-amino-4-methylpentanoic acid	C_6_H_13_NO_2_	131.17	*A. vera* and *A. saponaria*	Kim et al. (2013); Bajpai, (2018)
42	Isoleucine	2-amino-3-methylpentanoic acid	C_6_H_13_NO_2_	131.17	*A. vera, A. saponaria, and*	Kim et al. (2013); Martínez-Sánchez et al. (2020)
43	Phenylalanine	2-amino-3-phenylpropanoic acid	C_9_H_12_NO_2_	165.19	*A. vera,* and *A. saponaria*	Kim et al. (2013); Martínez-Sánchez et al. (2020)
44	Serine	2-amino-3-hydroxypropanoic acid	C_3_H_7_NO_3_	105.09	*A. vera, A. saponaria,* and *A. arborescens*	Kim et al. (2013); Mahor and Ali, (2016)
45	Threonine	2-amino-3-hydroxybutanoic acid	C_4_H_9_NO_3_	119.12	*A. vera* and *A. saponaria*	Kim et al. (2013); Martínez-Sánchez et al. (2020)
46	Tyrosine	2-amino-3-(4-hydroxyphenyl)propanoic acid	C_9_H_11_NO_3_	181.19	*A. vera* and *A. saponaria*	Kim et al. (2013); Martínez-Sánchez et al. (2020)
47	Cysteine	2-amino-3-mercaptopropanoic acid	C_3_H_7_NO_2_S	121.16	*A.vera*	Mahor and Ali (2016)
48	Methionine	2-amino-4-(methylthio)butanoic acid	C_5_H_11_NO_2_S	149.21	*A. vera*	Bajpai, (2018); Mahor and Ali, (2016)
49	Asparagine	2-amino-3-carbamoylpropanoic acid	C_4_H_8_N_2_O_3_	132.12	*A.vera*	Mahor and Ali (2016)
50	Glutamine	2-amino-4-carbamoylbutanoic acid	C_5_H_10_N_2_O_3_	146.14	*A. vera*	Mahor and Ali (2016)
51	Tryptophan	2-amino-3-(1H-indol-3-yl)propanoic acid	C_11_H_12_N_2_O_2_	204.22	*A. vera*	Martínez-Sánchez et al. (2020); Sotelo et al. (2007)
52	Aspartic acid	2-aminosuccinic acid	C_4_H_7_NO_4_	133.10	*A. vera, A. saponaria, A. arborescens* and *A. barbadensis*	Kim et al. (2013); Ahluwalia et al. (2022)
53	Glutamic acid	2-aminopentanedioic acid	C_5_H_9_NO_4_	147.13	*A. vera, A. saponaria, A. arborescens*	Kim et al. (2013); Mahor and Ali, (2016)
54	Lysine	2,6-diaminohexanoic acid	C_6_H_14_N_2_O_2_	146.19	*A. vera, A. saponaria* and *A. arborescens*	Kim et al. (2013); Mahor and Ali, (2016)
55	Arginine	2-amino-5-guanidinopentanoic acid	C_6_H_14_N_4_O_2_	174.20	*A. vera* and *A. saponaria*	Kim et al. (2013); Mahor and Ali, (2016)
56	Histidine	2-amino-3-(1H-imidazole-4-yl) propanoic acid	C_6_H_9_N_3_O_2_	155.15	*A. vera* and *A. saponaria*	Kim et al. (2013); Mahor and Ali, (2016)
57	Proline	pyrrolidine-2-carboxylic acid	C_5_H_9_NO_2_	115.13	*A. vera. A. saponaria* and *A. arborescens*	Kim et al. (2013)
58	Hydroxyproline	3-hydroxypyrrolidine-2-carboxylic acid	C_5_H_9_NO_3_	131.13	*A. vera*	Bajpai (2018)
59	Pyroglutamic acid	5-oxopyrrolidine-2-carboxylic acid	C_5_H_7_NO_3_	129.11	*A. barbadensis*	Ahluwalia et al., 2022
60	α-amino butyric acid	2-aminobutanoic acid	C_4_H_9_NO_2_	103.12	*A. vera* and *A. arborescens*	Kim et al. (2013)
61	β-alanine	3-aminopropanoic acid	C_3_H_8_N_2_O_4_	136.11	*A. vera, A. saponaria* and *A. arborescens*	Kim et al. (2013)
62	β-amino isobutyric acid	3-amino-2-methylpropanoic acid	C_4_H_9_NO_2_	103.12	*A. vera* and *A. saponaria*	Kim et al. (2013)
63	γ-aminobutyric acid (GABA)	4-aminobutanoic acid	C_4_H_9_NO_2_	103.12	*A. vera, A. saponaria, A. arborescens,* and *A. barbadensis*	Kim et al. (2013); Ahluwalia et al. (2022); Martínez-Sánchez et al. (2020)

### 3.7 Other amines of *Aloe* species

**FIGURE 8:**
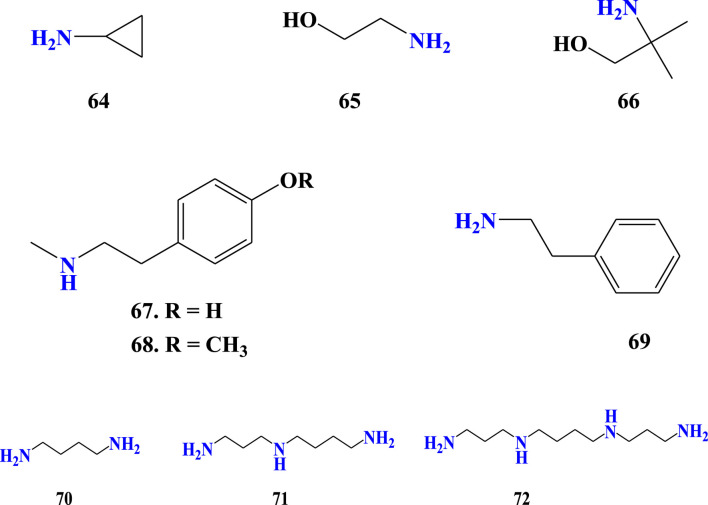
Amines of *Aloe* species.

Compounds 64–72 were reported from *Aloe* species (Figure 8). These compounds have a good sense if kept as amines rather than grouping them into other organic compounds such as alkloids. For instance, compounds (67) and (68) were reported as alkaloids from *Aloe* species (Dagne et al., 2000; Cock, 2015). The amine called 2-phenylethanamine (69) is a precursor for many compounds, including noradrenaline (Solomons, 2011), the hormone reported from *A. barbadensis* Mill. Regarding the amines detected from *Aloe* species, any amine may be monoamine or polyamine such as diamines, triamines, tetramines, or, etc., based on the number of nitrogens present in the structure (Table 4; Figure 8). Monoamines have one nitrogen in their structure, while polyamines have two or more ntrogens in their structures. The quantities of putresine, spermidine, and spermine polyamines have been analyzed from the leaf gels of *A. arborescens* Mill., *A. aristata* Haw., *A. claviflora* Str., *A. ferox* Mill., *A. mitriformis* Mill., *A. saponaria* Ait., *A. striata* Haw., and *A. vera* L, *A. barbadensis* Mill. (Beppu et al., 2004; Zapata et al., 2013; Ahluwalia et al., 2022). These polyamines have biochemical relationships. They also have specific odors, which make amines have unique odors, such as rotting fish. For instance, 1,4-butanediamine (**71**) has the appalling odors that might be expected from its common name, ‘putrescine’ derived from odor. Biologically, putrescine is synthesised from the catabolism of proteins (Mcmurry, 2016).

**TABLE 4:** Amines detected from *Aloe* species.

No	Name	Detection methods	Amine type based on number of N	Molecular formula	Molecular wight (g/mol)	References
64	Cyclopropanamine	GC-MS	monoamine	C_3_H_7_N	57.09	Ahluwalia et al. (2022)
65	2-aminoethanol	GC-MS	monoamine	C_2_H_7_NO	61.08	Ahluwalia et al. (2022); Kim et al. (2013)
66	2-amino-2-methylpropan-1-ol	GC-MS	monoamine	C_4_H_11_NO	89.14	Ahluwalia et al. (2022)
67	4-(2-(methylamino)ethyl) Phenol	-	monoamine	C_9_H_13_NO	151.21	Dagne et al. (2000)
68	2-(4-methoxyphenyl)-N-methylethanamine	-	monoamine	C_10_H_15_NO	165.24	Dagne et al. (2000)
69	2-phenylethanamine	GC-MS	monoamine	C_8_H_11_N	121.18	Ahluwalia et al. (2022)
70	Putrescine	HPLC-DAD, Spectrofluorometric detector fitted to HPLC	diamines	C_4_H_12_N_2_	90.14	Ahluwalia et al. (2022); Zapata et al. (2013); Beppu et al. (2004)
71	Spermidine	HPLC-DAD, Spectrofluorometric detector fitted to HPLC	triamines	C_7_H_19_N_3_	145.25	Zapata et al. (2013); Beppu et al. (2004)
72	Spermine	HPLC-DAD, Spectrofluorometric detector fitted to HPLC	tetraamines	C_10_H_26_N_4_	202.34	Zapata et al. (2013); Beppu et al. (2004)

“-” unspecified.

## 4 Biological activities of *Aloe* amines

Traditionally, people use various parts of *Aloe* species such as leaf gel, leaf latex, fresh leaf, root, flowers, etc. solely or by incorporating them into other substances for impotency in men, wounds, malaria, ticks, bloat and fire burn, caught, stomach ache, gonorrhea, swollen foot, strain, ascariasis, anthrax, internal parasite, weaning a child from breastfeeding, psychiatric disease, sprain, diabetes, liver disease, eye aliments, used as a poison, abdominal cramp, pasterlosis, black leg, skin softening, tuberculosis, and antiworms (Oda and Erena, 2017; Belayneh et al., 2020). These applications of *Aloe* species arise from the biologically active properties of the plants, which are due to their compounds (Radha and Laxmipriya, 2015; Mikayoulou et al., 2021). In modern days, the *in vivo* and *in vitro* bioactivities of *Aloe* species have been investigated. antioxidants (Hu et al., 2003; Lee et al., 2012; Sazhina et al., 2016), anti-inflammatory (Hajhashemi et al., 2012), antibacterial (Pandey and Mishra, 2010; Leitgeb et al., 2021), antifungal (Zapata et al., 2013), antiviral (Glatthaar-Saalmüller et al., 2015), antimalarial (Yadeta, 2022), anticancer (Karpagam et al., 2019), antidiabetic (Kazeem et al., 2022), wound healing (Fox et al., 2017), and etc. (Salehi et al., 2018).

In most of the *Aloe* species, the synergistic effects of amine compounds were investigated. In the literature, amine compounds were one of the compounds detected by GC-MS in *A. vera* for antibacterial activities and antioxidant capacity (Nejatzadeh-Barandozi, 2013). The study is similar to the work of Botes et al. (2008) on *A. greatheadii* var. *davyana*. These studies show the biological activities of the *Aloe* plants and the synergistic effect of the compounds, which indicate that amines have a vital role in the biological activities of *Aloe* extracts. In another way, Ahluwalia et al. (2022) tested compounds of *A. barbadensis* Miller including amines by correlating them to the effect of human blood T cells. The amine compounds, such as glucosamine single effect was identified from *A. barbadensis* Miller. Such tests identify the highly effective components of the plant extracts as well as the amine compounds such as glucosamine. The biological activities of amines in *Aloe* species have been summarized in Table 5. Generally, researchers use three approaches to determine the biological activities of *Aloe* species. These are: (i) using the extracts of the plant without identifying the active constituents when testing the medicinal potential of the plant is necessary; (ii) testing the bioactivities of the plants’ compounds synergistically; and (iii) identifying the bioactivities of the plants’ compounds specifically.

**TABLE 5:** Biological activities of *Aloe* species amines.

Amine compound	Biological activities	References
Pyrrolo [3,2-d] pyrimidin-2,4 (1H,3H)-dione	anti-inflammatory, antitumor, antioxidant, antiviral, anti-HIV agents, antiasthmatic, and anticoagulant	Alghamdi et al. (2023)
2-methyl-5-phenyl- pyrrole	Antimicrobial, inti-inflammatory, and antitumor	Alghamdi et al. (2023)
Polyamines	Antifungal activities	Zapata et al. (2013)
Glucosamine	Human blood T cell activity	Ahluwalia et al. (2022)
Vitamin B_6_	Antioxidant	Malik and Zarnigar (2013)
Vitamin B_12_	Production of red blood cells, antioxidants	Gajendra and Shaique, (2016); Ezzat et al. (2021)
Folic acid	Develop new blood cells, antioxidants	Gajendra and Shaique, (2016); Ezzat et al. (2021)
Choline	Production of energy, amino acid metabolism and developing muscle mass, antioxidants	Gajendra and Shaique, (2016); Ezzat et al. (2021)
Amino acids	Wound healing, moisturizing effect, Anti-inflammatory, antitumor, and basic building blocks of proteins in the body and muscle tissues	Maan et al. (2018); Laneri et al. (2020); Upadhyay, (2018); Sahu et al. (2013)
Hormones	Wound healing and anti-inflammatory	Sahu et al. (2013)

## 5 Medicinal applications

### 5.1 Pharmaceutical applications

**FIGURE 9:**
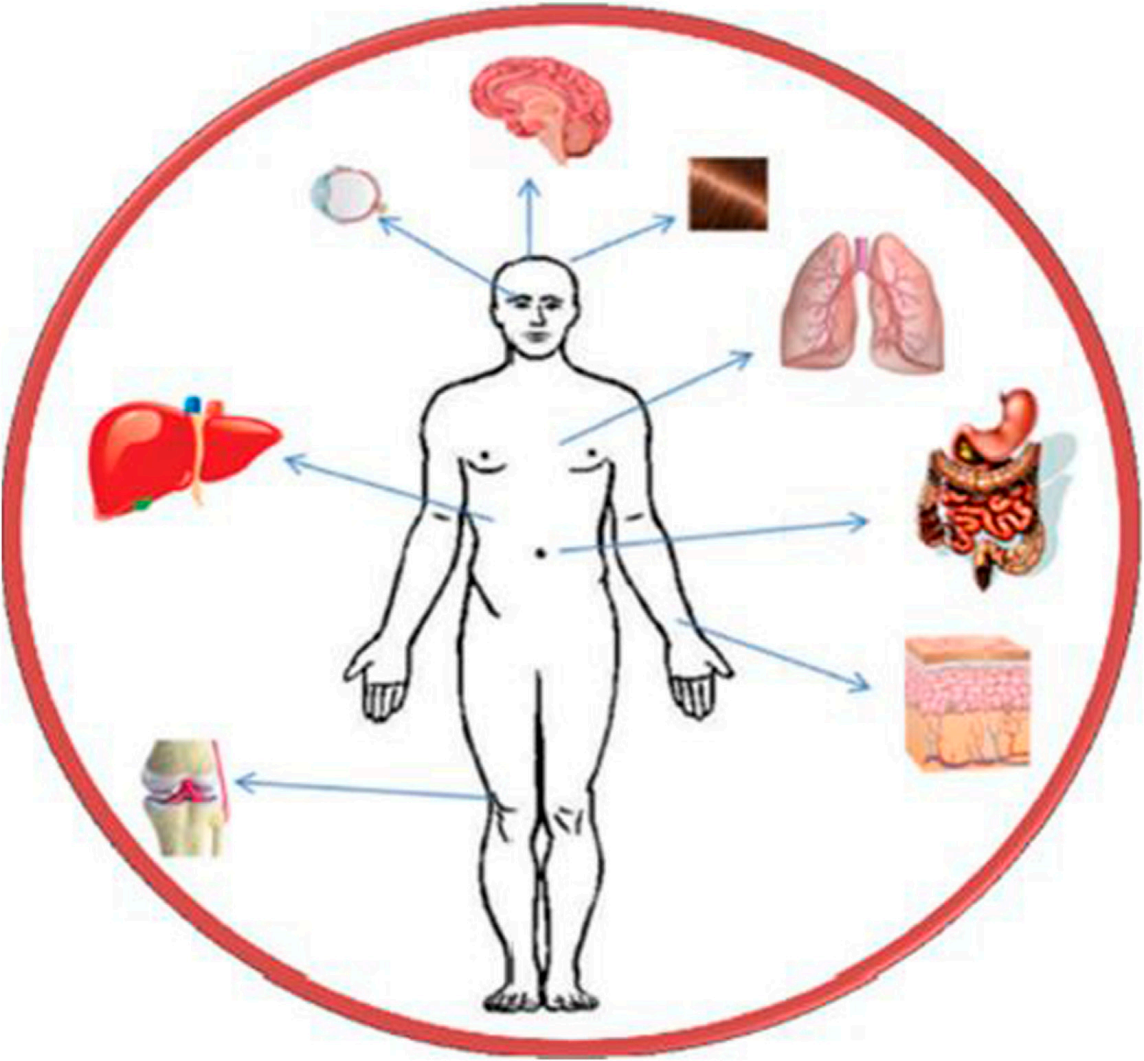
Specific mammalian body systems treated by therapeutic activities *Aloe* species.

The traditional uses of *Aloe* species have transformed into modern pharmaceutical applications. These days, studies on the biological activities of *Aloe* species both *in vivo* and *in vitro* verify the plant’s potential for particular mammalian body systems, including the brain, pancreas, liver, portal vein, intestine, muscles, tissues, lymphatic systems, and so on (Figure 9). In the literature, the therapeutic activities of *Aloe* species for the liver and kidney, gastrointestinal system, upper respiratory tract, reproductive (genital) organs, central and peripheral nervous systems, skin, eyes, hair, joints, and muscles have been reported (Akaberi et al., 2016). Other studies have used the healing properties of *Aloe* species to treat a variety of cancer diseases, including liver, colon, duodenal, skin, pancreatic, intestinal, lung, and kidney cancers (Singab et al., 2015). In addition, the metabolic disease Diabetes Mellitus (DM), commonly known as diabetes, has been treated by the medicinal effects of various *Aloe* species (Kumar et al., 2011; Chen et al., 2012). Based on these and other medicinal potentials of *Aloe* species, studies have shown that *A. vera* is used to make pharmaceutical products like ointments, tablets, and capsules (Eshun and He, 2004; Babu and Noor, 2020).

A large number of medically and biologically important compounds are amines. Because of their high degree of biological activity, many amines are used as drugs and medicines (Solomons and Fryhle, 2008; Wade, 2008). Alkaloids, amino acids, vitamins, and other amine compounds are biologically active compounds of *Aloe* species that are used in their medicinal applications and may lead to drug synthesis. For instance, 2-phenylethylamine, noradrenaline, and vitamins are mostly known amine compounds detected in *Aloe* species. Many phenylethylamine compounds have powerful physiological and psychological effects. Noradrenaline is a derivative of 2-phenylethylamine. Norepinephrine is hormone that is released into the bloodstream in response to stress. Recent evidence has elucidated significant changes in cerebral neurotransmitters in mice treated with *A. vera* extract, of which diminished levels of norepinephrine and serotonin are conspicuous (Solomons and Fryhle, 2008; Sultana and Najam, 2012). Norepinephrine is both a neurotransmitter and a hormone. It plays an important role in your body’s “fight-or-flight” response. As a medication, norepinephrine is used to increase and maintain blood pressure in limited, short-term, serious health situations (Mittal et al., 2017). Vitamins are essential for bodily functions such as helping to fight infection because of their biological properties such as wound healing, making our bones strong, and regulating hormones (Godswill et al., 2020). All vitamins detected in the *Aloe* species are water-soluble vitamins and function as coenzymes.

### 5.2 Nutraceutical applications

Several species of *Aloes* are mentioned in the literature for their applications as cooked vegetables, snack foods, famine foods, and preserve ingredients (Steenkamp and Stewart, 2007; Azaroual et al., 2012). *Aloes* are also utilized as food products and beverage ingredients due to their nutritional components combined to produce beneficial and biological effects (Sun et al., 2015). Owing to its advantageous characteristics in managing conditions like constipation, coughs, diabetes, headaches, arthritis, and immune system deficiencies, *Aloe* species gel is applicable in the food industry for functional foods (Minjares-Fuentes et al., 2016). Famous healthy foods developed from *A. vera* include dahi (a fermented South Asian dairy product) by replacing skim milk with *A. vera* gel, *A. vera* gel-enriched beverages, ice cream, lassi (a traditional fermented dairy beverage of South Asia), mango nectar, and carbonated beverages. Researchers have investigated the presence of bioactive compounds in such foods and beverages (Elbandy et al., 2014; Luo et al., 2022).

*A. vera* gel is used in the nutraceutical industry as a supplement in other food products and as a mineral source for a range of functional foods that are used to make different health drinks and beverages (Rodríguez et al., 2010; Ray et al., 2013). The food industry uses *A. vera* to make health drinks, jam, jelly, yogurt, cranberry, orange, grape, raspberry, pineapple, and strawberry beverages, among other functional goods (Kahramanoğlu et al., 2019; El-Sayed and El-Sayed, 2020). The *A. vera* plant has been reported for its antioxidant activities of nitrogen-containing vitamins and amino acids. Thus, the consumption of such dietary antioxidants from the *Aloe* species is beneficial in preventing cardiovascular diseases (Khanam and Sharma, 2013). Dried *A. vera* gel powder reduces body fat mass in diet-induced obesity rats, while its gel protects the liver from oxidative stress-induced damage in an experimental rat model. *A. vera* juice is marketed to support the health of the digestive system. *A. vera* is a good nutrition supplement for diabetic wound healing, while processed *Aloe* food products contain ingredients that show cancer prevention (Upadhyay, 2018).

Amino acids are required for the synthesis of body proteins and other important nitrogen-containing compounds (Yu and Fukagawa, 2020). Amino acid deficiency causes a number of disease states, nutritional deficiencies, fatigue, accelerated aging, and even premature death. Many pathological conditions, like a depressed immune system, weight loss, pressure sores, diarrhea, hair and skin depigmentation, and muscle weakness, are related to an amino acid deficiency (Shakerzadeh, 2016). Therefore, consuming *Aloe*-based products has nutraceutical applications. Especially essential amino acids can be obtained from food only. In another way, amino acid deficiency causes a number of disease states, nutritional deficiencies, fatigue, accelerated aging, and even premature death (Awuchi et al., 2020). Vitamins are other dietary nitrogen-containing compounds. Every nutrient that humans eat is on a mission to provide health benefits that support the pursuit of wellness. Vitamins work hard to keep our bodies functioning properly, and they help drive essential processes needed in our everyday lives (Tardy et al., 2020).

In another way, nutritional diseases such as cardiovascular disease, hypertension, cancer, and diabetes mellitus may arise from a nutrition deficiency like vitamins (Awuchi et al., 2020). In order to overcome the deficiencies of vitamins and health problems, consuming nutrition rich in vitamins plays a crucial role. Many diseases were treated with *Aloe* species (Salehi et al., 2018) since these plants have nutraceutical components like vitamins. Indirectly, people prevent and treat diseases and abnormal conditions when they consume these plants. This is because amine vitamins detected in *Aloe* species are among the water-soluble vitamins that are required for performing specific cellular functions, such as being precursors for coenzymes in the enzymes of intermediary metabolism (Alamgir, 2018). Table 6 summarizes the deficiency of amine vitamins (Kraemer et al., 2012; Griffiths, 2020; Taguchi, 2023) and treated diseases due to the presence of these vitamins, which have various biological effects (Channa et al., 2014; Akaberi et al., 2016; Belayneh et al., 2020; Ahluwalia et al., 2021; Sabbaghzadegan et al., 2021; Taqui et al., 2022).

**TABLE 6:** Amine containing vitamins from *Aloe* species and their nutraceutical applications.

Vitamins	Functions	Deficiency diseases	Symptoms	Diseases treated by *Aloe* species
Vitamin B_1_	Important in function of nervous system, helps release energy from foods, promotes normal appetite	Beriberi, Wernicke-Korsakoff syndrome	Anorexia, weight loss, weakness, peripheral neuropathy, gait ataxia, ophthalmoplegia, encephalopathy, dementia, and memory loss	Increasing appetite, central and peripheral nervous systems, treating tonsillitis, hematopoetic and immunomodulatory
Vitamin B_2_	Helps with vision, release energy from foods; healthy skin	Ariboflavinosis	Glossitis, cheilosis, dermatitis, growth retardation, conjunctivitis, and neuropathy	Anti-inflammatory, wound healing, central and peripheral nervous systems
Vitamin B_3_	Promotes healthy nerves, skin. Energy production from foods; aids digestion, and promotes normal appetite	Pellagra	Diarrhea, dematitis, dementia, and death	Treating diarrhea, wound healing, central and peripheral nervous systems
Vitamin B_6_	Aids in protein metabolism, absorption; aids in red blood cell formation; helps body use fats	dermatitis, anemia	Dermatitis, anemia, seizure, depression, encephalopathy, decline in immune function	Wound healing, hematopoetic and immunomodulatory effects
Vitamin B_9_	single carbon transfers	Anemia, neural tube defects	Diarrhea, depression, impaired cognition, and elevated risks of heart disease and stroke	Treating diarrhea, anti-fibrotic, anti-hypertensive, and anti-atherosclerotic
Vitamin B_12_	Metabolism of amino acids and fatty acids, DNA synthesis	Anemia	cognitive decline, Alzheimer’s disease, and vascular dementia	Treating of alzheimer’s disease, central and peripheral nervous systems
Choline	Helps brain and nervous system need it to regulate memory, mood, and muscle control	liver and muscle damage and increases in homocysteine	Liver disease, growth stunting, and immune dysfunction	Cardio protective effect, immunomodulatory effect

### 5.3 Cosmeceutical applications

**FIGURE 10:**
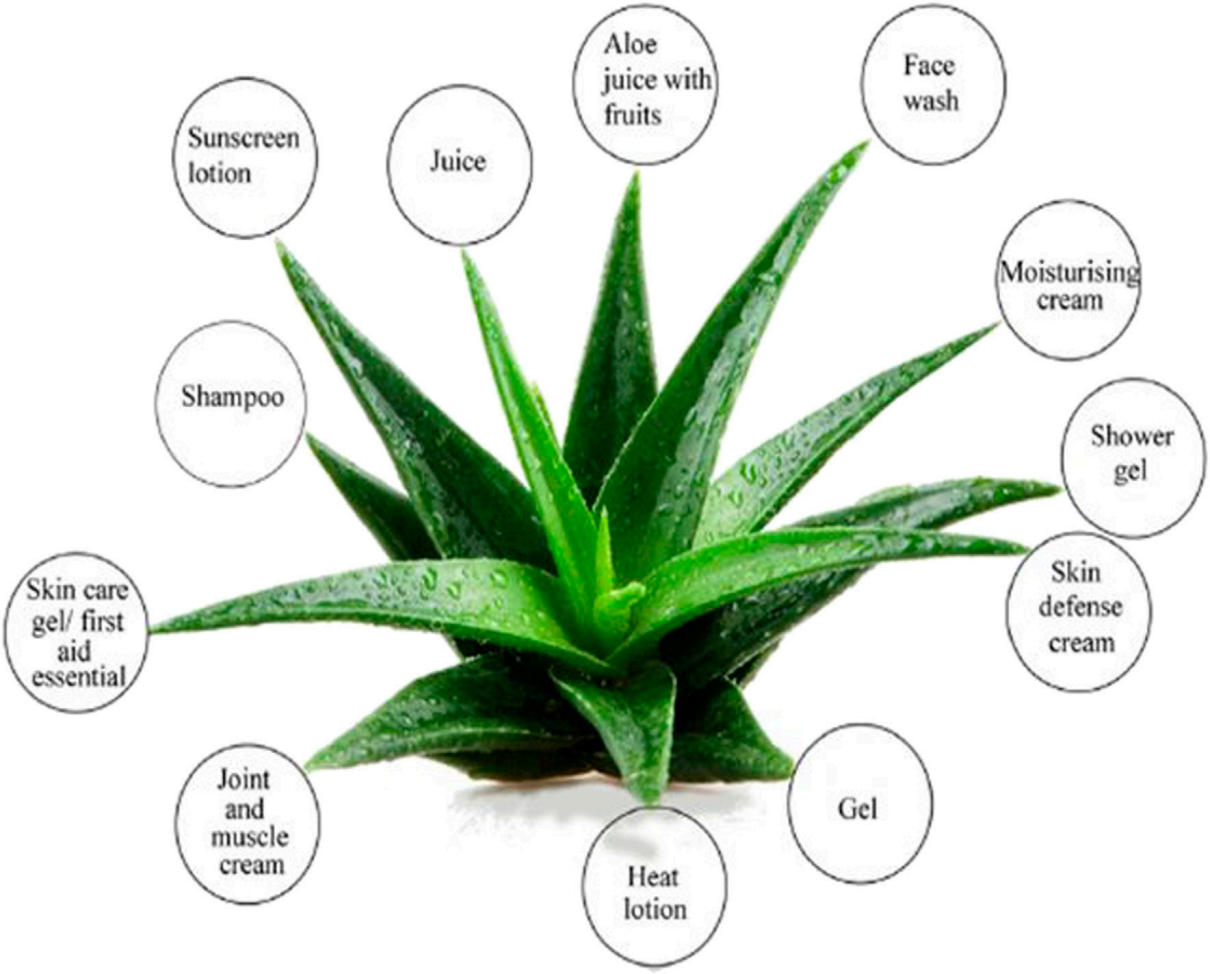
Some of *Aloe*-based cosmetics.

*Aloe* species are used in the preparation of traditional hair washing shampoos, which are transformed into industrial products in cosmetic and personal care (Sbhatu et al., 2020). *Aloes* ability to penetrate the epidermis, dermis, and hypodermis, expelling grease and bacteria from pores and inducing new cell production, which speeds up healing, is why they are used in cosmetics (de Rodriguez et al., 2006). Because of their medicinal potentials, *Aloe* species are incorporated into cosmetics and body care products such as soap, shampoo, *Aloe* bath gel, body wash, lotion, deodorant, lip balm, tooth gel, mouthwash, *Aloe* hand sanitizer, skin-replenishing agent, aloetic herbal beard oil, aloetic hair oil, under eye cream, conditioning face mask, skin toning cream, face scrub, shower gel, activator, exfoliator, detox capsules, and joint and muscle creams (Adlakha et al., 2022). One benefit of *Aloe*-based soaps is that they do not irritate skin or leave it feeling parched. *Aloe* extracts are also included in some shaving lotions and creams in the USA and Asia to speed up the healing of shaving wounds. In shaving creams, *A. vera* gel’s mucilaginous quality aids in its ability to act as a barrier of defense between the skin and beard (Lad and Murthy, 2013; Maan et al., 2018). Because of its high nutritional content and antioxidant qualities, *A. vera* is well known for its potent healing activity, even at the epithelial level of the skin. This results in the skin having a protective layer that speeds up healing. Ayurvedic medications for persistent skin conditions like psoriasis, acne, and eczema contain A. vera (Arunkumar and Muthuselvam, 2009; Aburjai and Natsheh, 2003). *Aloe* materials have been investigated for cosmeceutical applications due to their antioxidant activity of the polyphenols, indoles, alkaloids, amino acids like leucine and isoleucine, vitamins such as choline, cyanocobalamin, and folic acid that provide cleansing action and protection of photo-damages (Svitina et al., 2019; Ezzat et al., 2021). Some *Aloe*-based cosmetics are given in Figure 10.

## 6 Conclusion

Knowing the abundant components of *Aloe* species, one means of identification is very important, especially the components detected in a quantitative manner like amino acids and vitamins. The genus *Aloe* has various active compounds to prevent diseases when humans consume them since they have nutritional values and are used in cosmetics for their cosmeceutical values. In addition to these, various extracts of the *Aloe* have effective biological properties to treat diseases. For these reasons, the bioactive compounds of *Aloe* species should be studied in a comprehensive manner to provide direction for their medicinal potential. Especially identifications of medicinally active compounds like amine compounds have a great role in the development of drugs. Therefore, this trend is important for further studies on related topics. As a result, understanding the structure of amine compounds found in *Aloe* species provides scientific guidance for using the plants’ medicinal potential.
